# Comparative Quality of Laparoscopic and Open Cholecystectomy in the Elderly Using Propensity Score Matching Analysis

**DOI:** 10.1155/2010/490147

**Published:** 2010-12-22

**Authors:** Kazuaki Kuwabara, Shinya Matsuda, Koichi Benjamin Ishikawa, Hiromasa Horiguchi, Kenji Fujimori

**Affiliations:** ^1^Department of Health Care Administration and Management, Graduate School of Medical Sciences, Kyushu University, 3-1-1 Maidashi, Higashi-ku, Fukuoka 812-8582, Japan; ^2^Department of Preventive Medicine and Community Health, University of Occupational and Environmental Health, 1-1 Iseigaoka, Yahatanishi-ku, Kitakyushu 807-8555, Japan; ^3^Statistics and Cancer Control Division, National Cancer Center, 5-1-1 Tsukiji, Chuo-ku, Tokyo 104-0045, Japan; ^4^Department of Health Management and Policy, Graduate School of Medicine, The University of Tokyo, 7-3-1 Hongo, Bunkyo-ku, Tokyo 113-0033, Japan; ^5^Division of Medical Management, Hokkaido University, 5 Nishi 14 Kita, Kita-ku, Sapporo, Hokkaido 060-8648, Japan

## Abstract

The safety of laparoscopic cholecystectomy (LC) in patients ≥65 years of age requires further investigation of postoperative outcomes before it becomes more widely accepted as a safe technique. The advantages of using LC versus open cholecystectomy (OC) in elderly patients were analyzed using propensity score matching. The demographics, cholecystitis severity, comorbidities, complications, and admission and discharge Barthel Index (BI) scores of patients with benign gallbladder diseases were analyzed. Outcomes were analyzed by age, length of stay (LOS), total charges (TCs), BI improvement, and postoperative complications. OC, which was indicated in severe disease cases, increased hospital resource use and caused more complications than LC, but did not improve BI. Advanced age and OC resulted in greater LOS and TCs and was the best indicator of BI deterioration. Whenever possible, surgeons should use LC in elderly patients to minimize postoperative complications and allow them to regain a good quality of life.

## 1. Introduction

We live in an era of surgical innovation that has seen the development and expansion of various types of laparoscopic surgery in which the incisions made are increasingly small. It is well established that laparoscopic surgery, in comparison with more traditional methods, results in fewer post-operative complications and leads to earlier patient mobility and recovery of the normal activities of daily life [3, 4]. The safety of laparoscopic cholecystectomy (LC) for the elderly has also been confirmed in many studies as an acceptable procedure and is now the preferred method for cholecystectomy [1, 3–11].

Care-related outcomes such as mortality and post-operative complications have been studied extensively, but to the best of our knowledge, there have been no studies measuring functional changes in elderly patients undergoing cholecystectomies [1, 7–11]. The effects of age differences in elderly patients on the return to a good quality of life or on resource use also had not been previously or sufficiently investigated. Older patients are likely to have many chronic conditions that could impair their physical and functional recovery; it is important to monitor post-operative outcomes such as changes in activities of daily life [4, 8]. To complete the less-invasive LC procedure without conversion to open cholecystectomy (OC) in older patients, many surgeons will routinely use pre- or intraoperative bile duct interventions (BDIs), including endoscopic retrograde cholangiopancreatography (ERCP), intraoperative cholangiography (IOC), percutaneous gallbladder or common bile duct (CBD) drainage, endoscopic dilatation or sphincterotomy of the ampulla of Vater, and stone extraction from the CBD [12–16]. When compared with cases where LC was used without any interventional techniques, the use of added interventions can cause more stress to older patients and delay their recovery or worsen their physical condition; any evaluation of the safety of LC must include an evaluation of the type of intervention used. A few studies have demonstrated the safety of LC for older patients, and these were conducted in single centers or on a limited number of cases [1, 7, 9, 10]. They did not, however, measure the variation in patients' functional changes during hospitalization [9]. OC was reported to be used more than LC in seriously ill patients, regardless of age. If studies comparing the safety of LC to OC in older patients are to be done, they need to be randomized to eliminate any selection bias [7, 8, 11, 17].

Using a Japanese administrative database from fiscal year (FY) 2004 to 2008, we examined variation in the use of OC in patients ≥65 years of age. We analyzed the advantages of using LC instead of OC by propensity scoring in which we concurrently estimated the effects of age and OC on hospital resource use, postoperative complications and functional changes in recovering patients.

## 2. Methods

This retrospective study used both a Japanese administrative database and claim data that were incorporated into the Ministry of Health, Labour and Welfare (MHLW) database as well as our own research project that was designed to develop a Japanese case-mix classification system. Eighty-two academic and 1,346 community hospitals were enrolled in 2008. Anonymous health insurance claims data with detailed clinical information had been collected annually for this database for 4–6 months beginning July 1, 2002, and the information was provided to our research team. The database contained the date and quantity of care provided during hospitalization; therefore, it was used to assess hospital performance and payments [18]. 

Our database included a total of 8,010,361 possible patients from the 1,006 hospitals that have participated voluntarily in our research project from 2004 to 2008. In this group, we identified 13,709 cholecystectomy patients (11,677 LC and 2,032 OC) who were treated for benign gallbladder diseases in 122 hospitals participating in our project for five consecutive years. From the group of 13,709 cholecystectomy patients, 4916 were aged ≥ 65 and were enrolled in this study. Our project was approved by the ethical committee of the University of Occupational and Environmental Health, Fukuoka, Japan.

## 3. Variable Definitions

Study variables were as follows: age, sex, use of an ambulance (in an emergency situation), discharge outcome, discharge destination (to their home or other facility), presence of inflammation (as an indicator of the principal diagnosis), comorbidities, physical condition, and functional status at admission and discharge expressed by the Barthel Index (BI) score, biliary or procedure-related complications, use of IOC, pre- and/or postoperative ERCP or BDI, and hospital teaching status (community or academic hospital). 

Study patients were stratified into three age groups: 65–74, 75–84, and ≥85 years. Diagnoses were classified according to the International Classification of Disease 10th version (ICD code). A maximum of four comorbid conditions or four complications per patient were recorded in the database. To assess the severity of pre-existing comorbid conditions, we used the Charlson Comorbidity Index (CCI) [19]. Patients were divided into four groups and assigned a CCI of 0, 1, 2, ≥3. Using the ICD codes related to benign biliary disease (D135, K80–82), we categorized gallbladder status as follows: acute inflammation (K800, K803, K810 and K822) and chronic or other specified inflammation (K801, K804, K811–9, K820–1, K823, K830–2). The remaining ICDs were classified as no inflammation. Biliary or procedure-related complications included wound complications, hematoma or others (T81–T87), acute pancreatitis (K85), bowel obstruction (K560–7, K660 and K913), and peritonitis or intraabdominal abscess (K650–9) [20]. Academic hospitals were defined as university hospitals that were responsible for educating medical students and postgraduate trainees as well as carrying out clinical research.

Preoperative percutaneous gallbladder and CBD drainage, endoscopic dilatation and sphincterotomy of the ampulla of Vater, stone extraction, and stent insertion were classified as BDIs. ERCP was also examined as a factor independent of BDI. Patients who underwent conversion from LC to OC were classified as OC cases because of the lack of conversion information at this time. 

We calculated the operating room time; this time included induction of general anesthesia, insertion of the epidural anesthesia where applicable, preparation for video-monitoring, and extubation of the endotracheal tube as well as the skin-to-skin time. We also measured length of stay (LOS) and total charges (TCs) billed during admission. TC is considered to be a good estimate of healthcare costs [21]. The TCs included fees for physician consultation and administration, and costs of instruments, laboratory tests and imaging. The BI improvement score, often used as a quality of life indicator for the elderly, was also recorded. It was calculated as the BI score at discharge minus the score at admission; a negative BI score indicated a deterioration in BI score [2].

## 4. Statistical Analysis

Frequencies and proportions for categorical data for OC and LC cases were compared by Fisher's exact test. Continuous variables were compared using analysis of variance. The variations in LOS, TC, and the operating room time between OC and LC were also indicated in the box chart. Logistic regression was used to evaluate the OC-associated study variables. To reduce possible selection bias for cases indicating OC, we defined propensity score paired-matched cohorts and compared operating room time, resource use, and BI improvement score in each of the LC and OC groups [22]. Data from deceased patients were excluded and a mixed linear regression model was used to correlate age and OC with operating room time, LOS, TCs, and BI improvement score. In this model, every study hospital was treated as a random effect to control for independent hospital preferences for the type of cholecystectomy and hospital-specific standard practices. Logistic regression was used to evaluate the association of age and OC with complications and BI deterioration. Statistical analysis was performed using SPSS version 16.0, with a two-tailed level of significance set at *P* < .05.

## 5. Results

**Figure 1:**
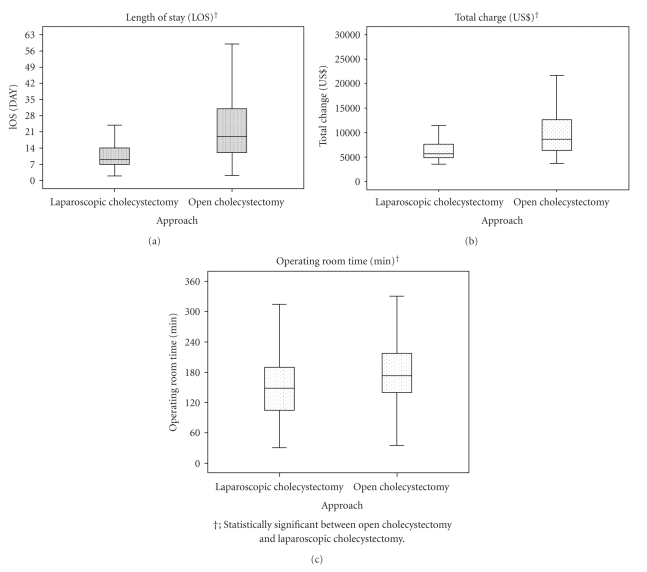
Resource use according to cholecystectomy approach.

Out of a total of 4,916 cholecystectomy patients, there were 3,692 LC patients from 122 hospitals and 1,224 OC patients from 117 hospitals. Of the LC patients, 1,071 (29.0%) were treated in 35 hospitals and 295 (24.1%) OC patients were treated in 34 academic hospitals. Twenty-one patients (two LC and 19 OC) were deceased and excluded. Older patients and those with greater CCI or acute gallbladder inflammation underwent OC more frequently. Preoperative ERCP or BDIs were performed more often in OC. Operating room time, LOS, TC, BI improvement score and complications were higher in OC, whereas BI scores at admission and discharge were lower (Table 1, Figure 1).

Advanced age (≥75 years), male sex, transport by ambulance to the hospital, presence of inflammation, and CCI of ≥2 were significant indicators for OC, but higher BI score at admission and surgery at an academic hospital was associated with less indication for OC (Table 2).

**Figure 2:**
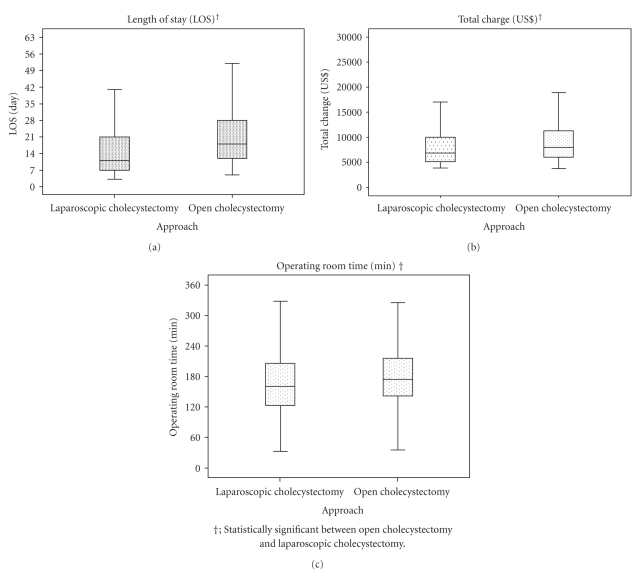
Resource use according to cholecystectomy approach after propensity score matching.

In the propensity score-paired matching cohorts, longer operating room time, longer LOS, and higher TCs were observed for OC, but the BI improvement score did not differ significantly between OC and LC for these parameters (Table 3, Figure 2).

Patients ≥75 years of age had longer LOS, and those ≥85 years of age had higher TCs. Patients between 75 and 84 years of age had a lower BI improvement score. OC was significantly associated with longer operating room time and LOS, and higher TCs, but not with BI improvement score. Complications were associated with greater LOS, TCs and less BI improvement scores (Table 4).

A risk of complications was observed in OC [odds ratio (OR) 1.285; 95% confidence interval (CI): 0.927–1.782] and patients with CCI ≥ 3 [OR: 1.894; 95% CI: 1.001–3.583]. The risk of complications was not related to age or BI at admission. Age, CCI ≥ 3, and complications were associated with BI deterioration: 75–84 years [OR: 2.908; 95% CI: 1.369–6.173], ≥85 years [OR: 3.998; 95% CI: 1.261–12.678], CCI ≥ 3 [OR: 3.998; 95% CI: 1.364–11.717], and complications [OR: 3.729; 95% CI: 1.768–7.865]. OC was not an independent indicator of BI deterioration (Table 5). 

## 6. Discussion

The present study was conducted to compare the advantages of LC versus OC in older patients in relation to changes in physical condition and ability to function. OC was employed more often in patients ≥75 years of age and those with greater CCI or the presence of gallbladder inflammation. Preoperative ERCP or BDIs were required more often in OC. Multivariate analysis of the propensity score matching cohorts revealed that LC had the advantage of fewer complications, shorter LOS, and lower TCs compared with OC. Operating room time and resource use were greater in OC. Neither cholecystectomy procedure caused significant variations in BI improvement scores or BI deterioration. Age did not determine the complications, but advanced age and complications were independent indicators of the functional recovery.

We observed an age disparity in the use of OC and LC in the older patients. Compared with the 75–84 year-old group, OC was used more frequently than LC for patients ≥85 years of age. This corresponds to the findings of a Swedish community study of cholecystectomies by Rosenmüller et al. [8]. We found that acute admission and perioperative use of ERCP were indicated more in OC; these results also agree with the findings of Rosenmüller et al. [8]. Generally, OC is considered for seriously ill and older patients as indicated in this study in which it was reported that BI at admission was lower in OC [8, 11]. These findings might cause a selection bias for OC that could exaggerate the benefits of laparoscopic surgery over conventional surgery.

Previous studies have confirmed the advantage of LC only in terms of mortality and complications; our study adds new and additional information concerning post-operative recoveries. In an aging population, major care-related outcomes related to the change in activity of daily life should also be considered. Kugler et al. estimated the functional recovery by measuring the change in BI score combined with information from the Hessian Stroke Data Bank [2]. In this study, however, the BI score was originally analyzed as an ordinal variable as opposed to a continuous one. The difference between ages 55 and 65 was not equivalent to that between ages 25 and 35; therefore, logistic regression needed to be applied to measure the association of OC with deterioration in BI score to correct for this discrepancy.

Since a randomized study for the elderly might be difficult to perform and unethical in that it would also depend on patient comorbidities, we constructed the propensity score matching cohorts using the administrative database. This kind of study might help surgeons make the right decision as to which factors are associated with functional recovery as well as which procedure is best for the elderly patient in actual clinical situations. Knowledge of functional changes would also contribute to the determination of healthcare policies for the elderly in the medically advanced G7 countries, where an ever-increasing number of aging patients require expensive surgical innovations [23]. The use of laparoscopic surgery should be favored because older patients are expected to benefit from fewer post-operative complications and/or earlier functional recovery. 

The extra costs of surgical innovations that promote earlier functional recovery would be offset by the overall benefits to the healthcare system derived from reducing LOS. Those costs should incorporate additional procedures such as the study BDIs that allow for the completion of laparoscopic surgery without conversion to OC. We attempted to address the concerns about treatment options for elderly patients by overcoming any selection bias as comprehensively as possible. Preoperative BDI, which is an indicator of disease severity, does not always appear to influence post-operative functional status, but the occurrence of post-operative complications appears to be a factor influencing functional recovery. As complications were not necessarily correlated with age, the key to achieving favorable outcomes in elderly patients is to manage post-operative complications [10]. OC and acute cholecystitis were also observed to be independent predictors of complications. Surgeons should choose the most appropriate surgical method to encourage the best possible post-operative recoveries for elderly patients. Clinical experts should also develop an educational program or model for teaching LC operating skills for cases in which more complicated gall bladder problems such as gallbladder inflammation are diagnosed [24]. 

Several limitations to our study should be discussed. The study period was limited to 4 months which might diminish our ability to generalize our results. However, our larger sample size and use of propensity scoring appeared to improve the validity of this study. Because the MHLW extended the study period to 12 months from FY 2010, use of this extensive database will expand the number of cases analyzed and overcome the initial limitation. A second point is that the duration of hospitalization in Japan is generally 2-3 times longer than that in Western countries [23]. Japanese hospitals generally provide wound management and nursing home services in addition to acute medical care; costs would reflect this type of care [25]. A third point that requires discussion is that some clinical information about conversion from LC to OC and the timing of the cholecystectomy were not included. OC cases in our study included those whose surgeries had been converted from LC to OC because the Japanese procedure codes do not define this conversion. However, it has been reported by Wolf et al. that the complications and LOS are similar between straightforward OC cases and those whose surgeries were converted from LC; our results therefore would not have been significantly distorted [11]. The last point to discuss is that the frequency of the procedure might have an effect on the quality of cholecystectomy care, physical condition, and functional status with emphasis on the residual respiratory function in fragile older patients [26]. Our administrative database should provide answers to these concerns because it has the quantity and date of use of every medical care item entered into it.

## 7. Conclusions

Our study investigated the quality of cholecystectomy care in patients ≥65 years of age using an administrative database and propensity score-paired matching analysis. The mix of patient cases explained the variation in use of cholecystectomy. After correcting for selection bias and relevant covariates, the LC method remained advantageous over OC in terms of fewer complications and lower resource use. Patients' functional status was not influenced by the type of cholecystectomy, but was affected by advanced age and complications. Surgeons should use LC in the elderly as much and as prudently as possible by planning the necessary preoperative treatment strategy and obtaining the necessary skills to complete LC without conversion to OC.

## Figures and Tables

**Table 1:** Patient characteristics, care process, and outcomes according cholecystectomy procedure (*n*, %).

		Aged 65 years or more		
		Laparoscopic cholycystectomy (3692)	Open cholecystectomy (1224)	*P*

Number of patients; community, academic		2621, 1071	929, 295	
Number of hospitals; community, academic		87, 35	83, 34	
Age	Mean	72.7 [5.6]	75.2 [6.5]	
	65–74 years	2461 (66.7)	619 (50.6)	<.001
	75–84 years	1104 (29.9)	485 (39.6)	
	≥85-years	127 (3.4)	120 (9.8)	
Sex				
	Male	1706 (46.2)	741 (60.5)	<.001
Ambulance				
	Used	120 (3.3)	172 (14.1)	<.001
Outcome				
	Mortality	2 (0.1)	19 (1.6)	<.001
Destination				
	not at home	101 (2.7)	97 (7.9)	<.001
Severity				
	Acute	493 (13.4)	520 (42.5)	<.001
	Chronic or others	1168 (31.6)	405 (33.1)	
Charlson comorbidity index				
	1	670 (18.1)	262 (21.4)	<.001
	2	272 (7.4)	152 (12.4)	
	3	87 (2.4)	74 (6.0)	
Preoperative ERCP only		131 (3.5)	67 (5.5)	.003
Preoperative BDI		372 (10.1)	270 (22.1)	<.001
IOC		132 (3.6)	28 (2.3)	.028

Study complication		351 (9.5)	175 (14.3)	<.001
	Acute pancreatitis	35 (0.9)	14 (1.1)	
	Peritonitis	18 (0.5)	24 (2.0)	
	Bowel obstruction	4 (0.1)	8 (0.7)	
Change of BI				
	Deterioration	55 (1.5)	28 (2.3)	<.001
	No change	2669 (72.3)	741 (60.5)	
	Improvement	130 (3.5)	123 (10)	

Teaching status				
	Academic	1071 (29)	295 (24.1)	.001

BI at admission		95.6 [17.1]	86.5 [29.9]	<.001^†^
BI at discharge		96.5 [15.4]	90.9 [24.7]	<.001^†^
BI improvement		0.9 [8.4]	4.4 [20.2]	<.001^†^

^†^Compared using analysis of variance. Other comparisons made using Fisher's exact test

[ ] standard deviation. C: community. A: academic. ERCP: endoscopic retrograde cholangiopancreatography, BDI: bile duct intervention. IOC: intraoperative cholangiography

**Table 2:** Variables associated with indications of open cholecystectomy (OC).

	Odds ratio	[95% CI]
Age (65–74 years)		
75–84 years	1.481	[1.242–1.767]
≥85-years	2.446	[1.702–3.514]
Gender		
Male	1.823	[1.538–2.159]
Ambulance		
used	2.257	[1.619–3.145]
Severity (for no inflammation)		
Acute	4.718	[3.78–5.889]
Chronic	1.929	[1.582–2.352]
Charlson comorbidity index (for zero)		
1	1.235	[0.998–1.527]
2	2.079	[1.586–2.724]
3	2.085	[1.355–3.209]
BI at admission	0.993	[0.989–0.997]
Preoperative ERCP	1.408	[0.965–2.056]
Preoperative BDI	1.197	[0.946–1.514]
Teaching status (for community)		
Academic	0.757	[0.619–0.926]

Hosmer Lemeshow goodness of model fit.	0.339	

***not included in the regression model. CI: confidence interval. BDI: bile duct intervention

ERCP: endoscopic retrograde cholangiopancreatography

**Table 3:** Patient characteristics, care process, and outcomes according to cholecystectomy procedure after propensity score matching (*n*, %).

		Laparoscopic cholecystectomy (775)	Open cholecystectomy (775)	*P*
Total		616(C), 159(A)	610(C), 165(A)	
Number of hospitals; community, academic		77(C), 23(A)	79(C), 32(A)	

Age	Mean	73.9 [6.1]	74.1 [6]	.493^†^
	65–74 years	435 (56.1)	441 (56.9)	.856
	75–84 years	291 (37.5)	290 (37.4)	
	≥85-years	49 (6.3)	44 (5.7)	
Sex				
	Male	449 (57.9)	462 (59.6)	.502
Ambulance				
	Used	59 (7.6)	66 (8.5)	.514
Destination				
	not at home	25 (3.2)	44 (5.7)	.019
Severity				
	Acute	256 (33)	259 (33.4)	.966
	Chronic or others	294 (37.9)	289 (37.3)	
Charlson comorbidity index				
	1	169 (21.8)	162 (20.9)	.915
	2	97 (12.5)	91 (11.7)	
	3	37 (4.8)	39 (5)	
Preoperative ERCP		55 (7.1)	44 (5.7)	.253
Preoperative BDI		153 (19.7)	147 (19)	.700
IOC		15 (1.9)	17 (2.2)	.721

Complication		75 (9.7)	93 (12)	.141
	Acute pancreatitis	9 (1.2)	7 (0.9)	
	Peritonitis	4 (0.5)	14 (1.8)	
	Bowel obstruction	3 (0.4)	3 (0.4)	
Change of BI				
	Deterioration	14 (1.8)	24 (3.1)	.054
	No change	692 (89.3)	662 (85.4)	
	Improvement	69 (8.9)	89 (11.5)	

Teaching status				
	Academic	159 (20.5)	165 (21.3)	.708

BI at admission		90.7 [24.2]	90.5 [24.9]	.852^†^
BI at discharge		93.3 [21]	93.6 [20.3]	.730^†^
BI improvement		2.6 [12.7]	3.2 [18]	.453^†^

^†^Compared using analysis of variance. Other comparisons were made using Fisher's exact test

[ ] standard deviation. C: community. A: academic. ERCP: endoscopic retrograde cholangiopancreatography

BDI: bile duct intervention. IOC: intraoperative cholangiography

**Table 4:** Variables associated with length of stay (LOS), total charge (TC), operating room time (min), and Barthel index (BI) improvement.

	LOS		TC		Operating room time		BI improvement	
	Estimation	95% CI	Estimation	95% CI	Estimation	95% CI	Estimation	95% CI
Intercept	11.6	[8.2–14.9]	6937	[5925–7949]	151.5	[132.7–170.3]	37.4	[34.0–40.9]
Age (for 65–74 years)								
75–84 years	2.9	[0.2–5.6]	620	[−196–1436]	1.2	[−13.1–15.4]	−6.1	[−8.8–−3.3]
85-years	1.9	[0.6–3.2]	483	[86–880]	−2.3	[−9.2–4.6]	−0.4	[−1.7–1.0]
Male	−0.1	[−1.4–1.2]	174	[−212–560]	6.0	[−0.7–12.7]	1.3	[0.0–2.6]
Ambulance	3.1	[0.8–5.5]	1487	[782–2193]	6.3	[−6.0–18.7]	3.1	[0.7–5.5]
Severity (for noinflammation)								
Acute	2.5	[0.9–4.1]	1032	[544–1519]	8.2	[−0.4–16.9]	−0.3	[−2.0–1.3]
Chronic or others	3.8	[2.1–5.6]	1690	[1167–2213]	18.3	[9.0–27.7]	0.8	[−1.0–2.6]
Charlson comorbidity index (for zero)								
1	7.3	[4.4–10.2]	2618	[1737–3499]	13.4	[−1.9–28.8]	−1.0	[−3.9–2.0]
2	2.3	[0.3–4.2]	799	[207–1391]	−4.8	[−15.1–5.6]	−0.9	[−2.9–1.1]
3	1.7	[0.1–3.3]	296	[−178–770]	−6.0	[−14.3–2.3]	0.4	[−1.2–2.0]
BI at admission, one point more	−0.046	[−0.073–−0.018]	−22	[−31–−14]	−0.013	[−0.161–0.135]	−0.388	[−0.416–−0.360]
Preoperative ERCP only	8.2	[5.7–10.8]	2147	[1371–2924]	8.6	[−5.0–22.2]	2.2	[−0.5–4.8]
Preoperative BDI	13.7	[12.0–15.3]	5480	[4979–5982]	12.0	[3.1–20.8]	0.9	[−0.8–2.6]
Approach (for laparoscopic cholecystectomy)								
Open cholecystectomy	7.9	[6.6–9.3]	1635	[1228–2041]	17.5	[10.1–24.9]	0.5	[−0.9–1.9]
IOC	0.2	[−4.3–4.7]	793	[−563–2149]	7.2	[−17.2–31.6]	−0.8	[−5.4–3.8]
Complication	4.7	[2.6–6.7]	1421	[806–2036]	***		−3.2	[−5.3–−1.1]
Teaching status (for community)								
Academic	0.6	[−1.7–2.9]	427	[−217–1071]	30.7	[15.5–46]	−1.5	[−4.0–1.0]

Akaike informationcriteria	12199		29920		17355		12272	

***not included in the regression model. CI: confidence interval. BDI: bile duct intervention.

ERCP: endoscopic retrograde cholangiopancreatography. IOC: intraoperative cholangiography.

**Table 5:** Variables associated with complications and Barthel index (BI) deterioration.

	Complication		BI deterioration	
	Odds ratio	[95% CI]	Odds ratio	[95% CI]
Age ( 65–74 years)				
75–84 years	1.232	[0.874–1.737]	2.908	[1.369–6.173]
≥85-years	1.147	[0.561–2.348]	3.998	[1.261–12.678]
Gender				
Male	0.877	[0.626–1.229]	0.430	[0.213–0.869]
Ambulance				
used	1.553	[0.916–2.633]	3.195	[1.326–7.700]
Severity (for no inflammation)				
Acute	1.665	[1.077–2.574]	0.952	[0.392–2.315]
Chronic	1.215	[0.786–1.877]	0.957	[0.410–2.234]
Charlson comorbidity index				
1	1.258	[0.848–1.866]	1.171	[0.490–2.799]
2	0.963	[0.555–1.671]	1.599	[0.567–4.512]
3	1.894	[1.001–3.583]	3.998	[1.364–11.717]
BI at admission	1.003	[0.996–1.010]	1.004	[0.99–1.017]
Preoperative ERCP	1.405	[0.779–2.533]	0.819	[0.185–3.632]
Preoperative BDI	0.923	[0.607–1.404]	0.605	[0.241–1.516]
Approach (for laparoscopic cholecystectomy)				
Open cholecystectomy	1.285	[0.927–1.782]	1.729	[0.871–3.432]
IOC	0.586	[0.137–2.508]	2.65	[0.572–12.279]
Complication	***		3.729	[1.768–7.865]
Teaching status (for community)				
Academic	1.989	[1.389–2.849]	0.899	[0.389–2.082]

Hosmer Lemeshow goodness of model fit.	0.266		0.810	

***not included in the regression model. CI: confidence interval. BDI: bile duct intervention. ERCP: endoscopic retrograde cholangiopancreatography. IOC: intraoperative cholangiography.
